# Upper gastrointestinal haemorrhage caused by duodenal metastasis from primary lung cancer: A case report

**DOI:** 10.1002/rcr2.1105

**Published:** 2023-02-14

**Authors:** Maissa Jellali, Mohamed Ali Chaouch, Sadok Ben Jabra, Amani Moussa, Hanene Zenati, Khadija Zouari, Faouzi Noomen

**Affiliations:** ^1^ Department of visceral and digestive surgery Monastir University Hospital Monastir Tunisia

**Keywords:** bleeding, duodenal metastases, haemorrhage, lung cancer

## Abstract

Duodenal metastases from primary lung carcinoma are uncommon. They usually occur in terminal‐stage disease. Bleeding, as the first presentation of duodenal metastases, is rare. This case reports a rare mechanism of upper gastrointestinal bleeding due to a metastatic involvement of the duodenum and gastroduodenal artery. A 58‐year‐old man with a past medical history of pulmonary carcinoma presented an episode of hematemesis of great abundance with melena. On physical examination, he was afebrile and pale. The biological data found an anaemia with haemoglobin at 6 g/dL. The upper gastrointestinal endoscopy revealed a congestive duodenal lesion with signs of recent bleeding. An angio CT scan localized the bleeding from the gastroduodenal artery. A few hours later, the patient presented a recurrent episode of hematemesis with deglobalization. So we performed a radiologic embolization of the gastroduodenal artery. Haemorrhage as the first presentation of small bowel metastases is rare, especially when these are located in the duodenum, with a poor prognosis. Radiological embolisation could be the best choice for treatment.

## Graphical abstract

An extremely rare case of upper gastrointestinal haemorrhage caused by duodenal metastasis from primary lung cancer.

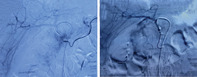

## INTRODUCTION

Duodenal metastases from primary lung carcinoma are uncommon. They usually occur in patients with terminal‐stage disease.^1^ These metastases are usually asymptomatic but may be revealed by perforation, obstruction, malabsorption, or bleeding.^2^, ^3^ Bleeding, as the first presentation of duodenal metastases, is rare and related to poor patient survival. We present a case of a 58‐year‐old patient with upper gastrointestinal bleeding due to duodenal metastases from a primary lung adenocarcinoma. This case aims to report a rare mechanism of upper gastrointestinal bleeding due to a metastatic involvement of the duodenum and gastroduodenal artery.

## CASE REPORT

**FIGURE 1:**
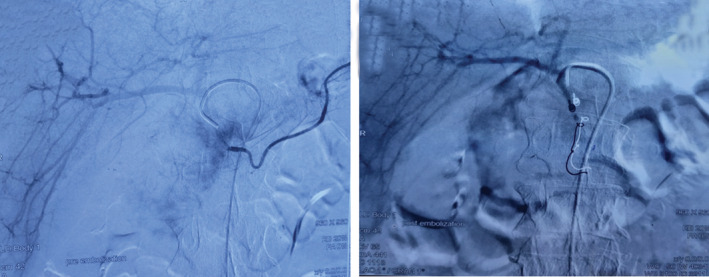
Radiologic angiography of the pre and post embolization of the gastroduodenal artery

A 58‐year‐old man, former smoker with a 20‐pack year history of smoking, and a past medical history of pulmonary carcinoma, presented to the Emergency Department with an alteration of the general state with a weight loss estimated at 20 kg in 2 months. He presented an episode of hematemesis with melena. On physical examination, he was afebrile and pale. The blood pressure was normal, with no tachycardia. The biological investigations found an anaemia with haemoglobin at 6 g/dL. The upper gastrointestinal endoscopy revealed a congestive duodenal lesion with signs of recent bleeding. So, we initiated resuscitation with blood transfusion with supervision in the intensive care unit. An angio CT scan was performed. It localized the bleeding from the gastroduodenal artery. A few hours later, the patient presented a recurrent episode of the hematemesis. So we proposed a radiologic embolization of the gastroduodenal artery (Figure 1). The post interventional follow‐up was marked by a stabilization of the haemodynamic parameters with no necessity of transfusion. The patient was examined 2 weeks later in the outpoint clinic and readdressed to the oncologist for a pulmonary carcinoma follow‐up.

## DISCUSSION

This case report highlights an uncommon complication of pulmonary carcinoma metastases discovered in front of upper gastrointestinal bleeding due to an involvement of the duodenum and gastrointestinal artery.

Lung cancer is one of the most common cancers worldwide and takes first place among the causes of death associated with cancer. The most common sites of metastases were the liver, adrenal gland, bone, and brain. In contrast, lung cancer metastasis to the gastrointestinal tract is rare, except oesophagus.^1^ The prevalence of small bowel metastases from lung cancer ranges between 2.6% and 10.7%, and most were found incidentally at autopsy examination in patients with advanced or widely disseminated lung cancer.^4^ Although any tumour cell type of lung cancer can develop gastrointestinal metastases, the most common type is a squamous cell, followed by a large cell, and then adenocarcinoma.^5^ Among small bowel metastasis, the jejunum is the most frequent site of involvement (50.9%), followed by the ileum (33.3%) and the duodenum (15.8%).^6^ Duodenal metastases from lung cancer are unusual. In a series of 15 patients with small bowel metastases from lung cancer by McNeill et al. none had duodenal metastasis.^2^ Duodenal metastases rarely show symptoms; however, duodenal involvement of lung cancer can elicit melena, hypochromic microcytic anaemia, upper gastrointestinal bleeding, malabsorption, intussusception, obstruction and perforation.^6^ Haemorrhage or bleeding from duodenal metastases is uncommon, as in our patient; however, there are a few reports of duodenal metastasis from primary lung cancer with melena. There are other reports of gastrointestinal bleeding from duodenal metastasis after lung cancer diagnosis.^4^ Duodenal metastasis poses a diagnostic dilemma, as radiologic imaging is often unremarkable, that is, this patient. In such cases, an endoscopic evaluation and biopsy should be performed to establish a definitive diagnosis, especially if the cause of melena or microcytic anaemia cannot be ascertained.^6^ On endoscopy, most metastatic lesions present as “volcano‐like tumours” or submucosal tumours with bridging folds and superficial ulcerations. Moreover, capsule endoscopy is more effective in detecting small bowel abnormalities when compared to push enteroscopy.^5^ In addition to the endoscopic examination, immunohistochemical (IHC) staining and histological analysis are keys to the diagnosis. It is often necessary to differentiate between different carcinomas or to distinguish a primary tumour from a metastatic tumour.^5^ The patient we report also had extensive metastasis at different sites, including liver and bilateral adrenal. Such extensive metastasis may be linked to the activation of different molecular pathways enabling epithelial‐mesenchymal transition, cell migration and invasion.^4^ The treatment of duodenal metastasis is also challenging, and it depends on the site of duodenal involvement, patient characteristics, and the size of these lesions. However, most cases require duodenectomy or pancreaticoduodenectomy (Whipple procedure) for symptomatic relief. Endoscopic resection of smaller duodenal metastatic lesions (≤1 cm) appears to be safe and effective, especially in cases in which these metastases may be removed completely by endoscopic methods.^6^ Due to the condition's rarity, the optimal management of bleeding duodenal metastasis is unknown. Endoscopic management may be attempted initially as it is effective.^2^ Surgical treatment was not recommended in this case due to the patient's comorbidities and metastatic disease; a palliative gastroduodenal artery embolization was ultimately performed with a good response. Palliative chemotherapy or radiotherapy to alleviate symptoms can be considered appropriate.^4^ The role of radiation therapy needs to be investigated, as only a case report utilizing radiation therapy for duodenal metastasis has been reported.^6^ Duodenal metastasis is associated with a dismal prognosis. The median survival of patients with intestinal metastasis is 23 months.^4^ Only a few cases have survived more than 12 months after surgical resection of duodenal metastases, except one patient who survived 46 months.^6^

In conclusion, duodenal metastases from primary lung carcinoma are extremely rare entities that usually occur in patients with a terminal disease and rarely produce symptoms. Haemorrhage as the first presentation of small bowel metastases is rare, especially when these are located in the duodenum, with a poor prognosis. Endoscopic resection can safely manage smaller lesions (≤1 cm).

## AUTHOR CONTRIBUTIONS

All the authors participated in the writing of the manuscript and validated the final version of the manuscript.

## CONFLICT OF INTEREST STATEMENT

None declared.

## ETHICS STATEMENT

The authors declare that appropriate written informed consent was obtained for the publication of this manuscript and accompanying images.

## Data Availability

Data sharing not applicable to this article as no datasets were generated or analysed during the current study
